# PYCR1 interference inhibits cell growth and survival via c-Jun N-terminal kinase/insulin receptor substrate 1 (JNK/IRS1) pathway in hepatocellular cancer

**DOI:** 10.1186/s12967-019-2091-0

**Published:** 2019-10-16

**Authors:** Juhua Zhuang, Yanan Song, Ying Ye, Saifei He, Xing Ma, Miao Zhang, Jing Ni, Jiening Wang, Wei Xia

**Affiliations:** 1 Department of Nuclear Medicine, The Seventh People’s Hospital of Shanghai University of Traditional Chinese Medicine, 358 Datong Road, Pudong, Shanghai, 200137 People’s Republic of China; 2 Central Laboratory, The Seventh People’s Hospital of Shanghai University of Traditional Chinese Medicine, 358 Datong Road, Pudong, Shanghai, 200137 People’s Republic of China

**Keywords:** PYCR1, Hepatocellular carcinoma, JNK, IRS1, Insulin resistance

## Abstract

**Background:**

Liver cancer is the second leading causes of cancer-related death globally. Pyrroline-5-carboxylate reductase 1 (PYCR1) plays a critical role in metabolic profiles of tumors. Therefore, it is necessary to explore the mechanisms of PYCR1 on cell growth and survival in hepatocellular carcinoma (HCC).

**Methods:**

Protein and mRNA expression levels of PYCR1 in 140 pairs of tumor and adjacent normal liver tissues of HCC patients were analyzed by immunohistochemistry and quantitative real-time polymerase chain reaction (qRT-PCR). Expressions of PYCR1 were inhibited in BEL-7404 cells and SMMC-7721 cells using gene interference technology. The cell proliferation was detected by Celigo and MTT assay. The colony formation assay was also performed. The cell apoptosis was measured by flow cytometric assay. The effect of PYCR1 interference on tumor growth was observed by xenograft nude mice assay in vivo. The downstream pathway of PYCR1 interference was searched by microarray and bioinformatics analysis, and validated by qRT-PCR and western blot.

**Results:**

PYCR1 levels were significantly up-regulated in HCC tumor tissues than adjacent normal liver tissues in both protein and mRNA levels (*P *< 0.01). In vitro, the cell proliferation was significantly slower in shPYCR1 group than shCtrl group in BEL-7404 and SMMC-7721 cells (*P *< 0.001). The colony number was significantly smaller after PYCR1 interference (*P *< 0.01). The percentage of apoptosis cells significantly increased in shPYCR1 group (*P *< 0.01). In vivo, PYCR1 interference could obviously suppress tumor growth in xenograft nude mice. The volume and weight of tumors were significantly smaller via PYCR1 interference. The c-Jun N-terminal kinase (JNK) signaling pathway significantly altered, and insulin receptor substrate 1 (IRS1) were significantly down-regulated by PYCR1 interference in both mRNA and protein levels (*P *< 0.001).

**Conclusion:**

PYCR1 interference could inhibit cell proliferation and promote cell apoptosis in HCC through regluting JNK/IRS1 pathway. Our study will provide a drug target for HCC therapy and a potential biomarker for its diagnosis or prognosis.

## Background

Liver cancer is the second leading causes of cancer-related death globally [1]. 90% of primary liver cancer patients belong to hepatocellular carcinoma (HCC) [2]. The incidence and mortality rates for HCC have greatly increased during the past two decades, and the majority of HCC patients occur in eastern/south-eastern Asia and Africa [3]. Although surgical hepatic resection and liver transplantation techniques have improved in recent years, the overall 5-year survival rate is still only around 5% and the long-term prognosis remains discouraging [4, 5]. Therefore, a better understanding of the key molecules and regulative pathways involved in the etiology and progression of HCC may lead to improved treatments.

Growing tumors alter their metabolic profiles to meet the biosynthetic and bioenergetics demands of increased cell growth and proliferation [6]. Many studies have focused on the metabolic regulatory roles of amino acids in cancers, especially the nonessential amino acids (NEAA) [7, 8]. Among these, the regulatory functions of proline metabolism proposed 3 decades ago have been emphasized [9]. On one hand, the pyrrolidine ring makes proline a unique proteogenic amino acid with a distinctive role in protein folding and secondary structures [10]. On the other hand, proline also plays key roles in many aspects, such as cellular signaling processes [11], cellular bioenergetics [12], and cancer cell metabolism [13]. Recent discoveries paid attention to the broad effects of proline metabolism on cancer cell growth and survival, which implicated proline metabolic enzymes as potential targets for therapeutic intervention [14–17]. Pyrroline-5-carboxylate reductase 1 (PYCR1) plays a critical role in proline biosynthesis and catalyzes the NADH-dependent conversion of pyrroline-5-carboxylate (P5C) to proline [18]. However, the role of PYCR1 in HCC cell growth and survival is still unclear.

In this study, we aim to analyze the relationship between PYCR1 and HCC, explore the role of PYCR1 regulating c-Jun N-terminal kinase/insulin receptor substrate 1 (JNK/IRS1) pathway, and clarify the mechanisms of PYCR1 affecting HCC cell growth and survival. The achievements will probably provide us a potential drug target for therapy or a biomarker for diagnosis and prognosis.

## Materials and methods

### Tissue specimens

The tumor and adjacent normal tissues used for immunohistochemistry were collected from 90 HCC patients who underwent surgery at Shanghai Seventh People’s Hospital between 2007 and 2009. The informed consent was obtained from every study participant. The study was conducted in accordance with the Declaration of Helsinki, and the study protocol was approved by the Institutional Research Ethics Committee in 2007 (No. 2007-019). The mRNA profiles used in the study were obtained from the Cancer Genome Atlas (TCGA) database (http://cancergenome.nih.gov).

### Immunohistochemistry (IHC)

Tissues were fixed in 10% buffered formalin, embedded in paraffin and cut into 4 μm sections. Deparaffinized sections were treated with methanol containing 3% hydrogen peroxide for 10 min before conducting antigen retrieval using a microwave oven at 95 °C for 5 min and cooling at 25 °C for 2 h. After washing with PBS, blocking serum was applied for 20 min. The sections were incubated with primary antibodies overnight at 4 °C and biotin-marked secondary antibodies for 20 min followed by a peroxidase-marked streptavidin for an additional 20 min. The reaction was visualized by using 3, 3′-diaminobenzidine tetrahydrochloride. The nuclei were counterstained with hematoxylin. Reproducibility of staining was confirmed by reimmunostaining via the same method in multiple, randomly selected specimens.

### Cell culture

The HCC cell lines used in this study included BEL-7404 cells and SMMC-7721 cells. They were obtained from the Type Culture Collection of the Chinese Academy of Sciences, Shanghai, China. BEL-7404 and SMMC-7721 cells were both maintained in 1640 medium (Gibco, Gaithersburg, MD, USA), supplemented with 10% fetal bovine serum (Gibco, Gaithersburg, MD, USA), 100 U/ml penicillin and 0.1 mg/ml streptomycin (Gibco, Gaithersburg, MD, USA) at 37 °C in 100% air.

### RNA interference

The sequences for RNA interference were 5′-TTCTCCGAACGTGTCACGT-3′ for control, and 5′- CAGTTTCTGCTCTCAGGAA-3′ for PYCR1 (GenePharma, Shanghai, China). The sequences were inserted into Age I and EcoR I points of GV115 vector. The shRNAs of GV-NC-GFP-shRNA and GV-PYCR1-GFP-shRNA were constructed as control lentivirus and shPYCR1 lentivirus, and positive clones were screened and identified. Subsequently, HCC cells were seeded in a 6-well plate at a density of 2 * 10^5^/well for RNA interference. According to the results of preliminary experiment, HCC cells were infected with a certain amount of control lentivirus and shPYCR1 lentivirus. At 72 h post-infection, the expression of reporter gene GFP was observed by the fluorescent microscope. The fluorescence rate was the positive infective rate. If the infective rate was larger than 70% and the state of cells was normal, the further study could be continued.

### Quantitative real-time polymerase chain reaction (qRT-PCR)

Total RNA from HCC cells was extracted using TRIzol Reagent (Invitrogen, Carlsbad, CA, USA). RNA concentration was determined by NanoDrop 2000 spectrophotometer (Thermo Fisher Scientific, Rockford, IL, USA). A total of 20–100 ng RNA was reverse-transcribed using First-Strand cDNA Synthesis kits (Invitrogen, Carlsbad, CA, USA) according to the manufacture’s protocol. qRT-PCR was performed on ABI 7500 System (Applied Biosystems, Foster City, CA, USA) and used to analyze the expression levels of mRNA. Relative expression level of genes was calculated using GAPDH as the internal control. Each sample was run three times. The primer pairs used in the study are listed as follows. PYCR1 forward: 5′-GGCTGCCCACAAGATAATGGC-3′; reverse: 5′-CAATGGAGCTGATGGT GACGC-3′; GAPDH forward: 5′-TGACTTCAACAGCGACACCCA-3′; reverse: 5′-CACCCTGTTGCTGTAGCCAAA-3′.

### Western blot

The cells were first washed with cold PBS three times, then lysed in RIPA lysis buffer (Beyotime, Shanghai, China) containing a complete protease inhibitor cocktail (Boehringer Mannheim, USA), and cell lysates were collected. Concentrations of proteins were then determined by bicinchoninic acid (BCA) protein assay kit (Boster, Wuhan, China). About 20–50 μg of proteins were separated by 10% SDS-polyacrylamide gel and then transferred to a polyvinylidene difluoride (PVDF) membrane. Subsequently, the membrane was incubated with primary antibodies against PYCR1 (1:200) (Abcam, UK), JUN (1:500) (Abcam, UK), IRS1 (1:500) (Abcam, UK) and GAPDH (1:2000) (Santa Cruz, Dallas, TX, USA) overnight at 4 °C, and appropriate secondary antibodies (1:2000) (Santa Cruz, Dallas, TX, USA) for 1 h at room temperature. Signals were detected by gel imaging system (ProteinSimple, San Jose, CA, USA). The ratio of interested proteins to GAPDH was analyzed by FluorChem FC3 software. Western blot experiments were performed in triplicate.

### Cell proliferation assay

HCC cells infected with control lentivirus (2.5 μl, 8 × 10^8^ TU/ml) and shPYCR1 lentivirus (6.7 μl, 3 × 10^8^ TU/ml) were seeded in 96-well plates at a density of approximately 2–5 * 10^3^/well in quintuplicate. Cell proliferation was performed at specific times, such as 1st day, 2nd day, 3rd day, 4th day and 5th day. The fluorescence was measured to access cell counts using Celigo Imaging Cytometer (Nexcelom Bioscience, Lawrence, MA, USA). In addition, for MTT assay, 20 μl of MTT solution was added to each well. After 4 h incubation at 37 °C, the solution was removed and DMSO was added to dissolve the formazan crystals formed. The absorbance at 490 nm was measured Glomax-Multi + Detection System (Promega, Madison, WI, USA). At least three independent experiments were performed.

### Colony formation assay

Cells were seeded in 6-well plates at a concentration of 400–1000 cells per well and changes culture with media every 72 h. Colonies were fix with 4% paraformaldehyde and stained with GIEMSA solution (Dingguo, Shanghai, China) after 14 days, then colonies were visualized by fluorescence microscope and quantitated. At least three independent experiments were performed.

### Cell apoptosis assay

Apoptosis assay was performed using FITC-Annexin V Apoptosis Detection Kit (BD Biosciences, Franklin Lakes, NJ, USA) according to the manufacture’s instruction. Briefly, the cells were collected by trypsin, washed twice with ice-cold PBS and resuspended in 1 * binding buffer. Then, 10 μl of annexin V-FITC and propodium were added into 100 μl of cell suspensions. After incubation for 15 min, the samples were analyzed using the Beckman Counter flow cytometer.

### In vivo tumor growth experiment

A total of twenty female BALB/C nude mice (4–6 weeks, 18–22 g) were purchased from Shanghai Lingchang Biotechnology (SCXK2013-0018). All of them were randomly separated into two groups, NC group (n = 10) and KD group (n = 10). They were subcutaneously inoculated with SMMC-7221 cells (5 * 10^6^) stably expressing control lentivirus and shPYCR1 lentivirus, respectively. After the diameter of tumor was larger than 5 mm, tumor volume was determined twice per week. The tumor volume was calculated as V = (width^2^ * length)/2. The bioluminescence imaging was detected before sacrifice. Tumor was obtained and weighed after sacrifice. All animal studies were approved by the Ethics Committee of Shanghai Seventh People’s Hospital.

### Microarray and bioinformatics analysis

Total RNA was extracted from HCC cells using Trizol reagent in shCtrl group (n = 3) and shPYCR1 group (n = 3). The qualified samples were analyzed on Affymetrix GeneChip primeview human gene expression array. Differentially expressed genes were selected with a cut-off *P* value of less than 0.05 based on statistical analysis and a twofold change cut-off value. Those differentially expressed genes obtained from the microarray analyses were uploaded to Ingenuity Pathway Analysis (IPA, Ingenuity Systems) and a core biologic pathway analysis was performed to identify molecular pathways.

### Statistical analysis

Statistical analysis was performed by SPSS 16.0 (Chicago, IL, USA). The data were expressed as mean ± standard deviation and analyzed using Mann–Whitney test because of abnormal distribution or heterogeneity of variance. *P *< 0.05 was considered statistically significant. All results were confirmed by at least three independent experiments.

## Results

### PYCR1 was over expressed in HCC tumor tissues

**Fig. 1:**
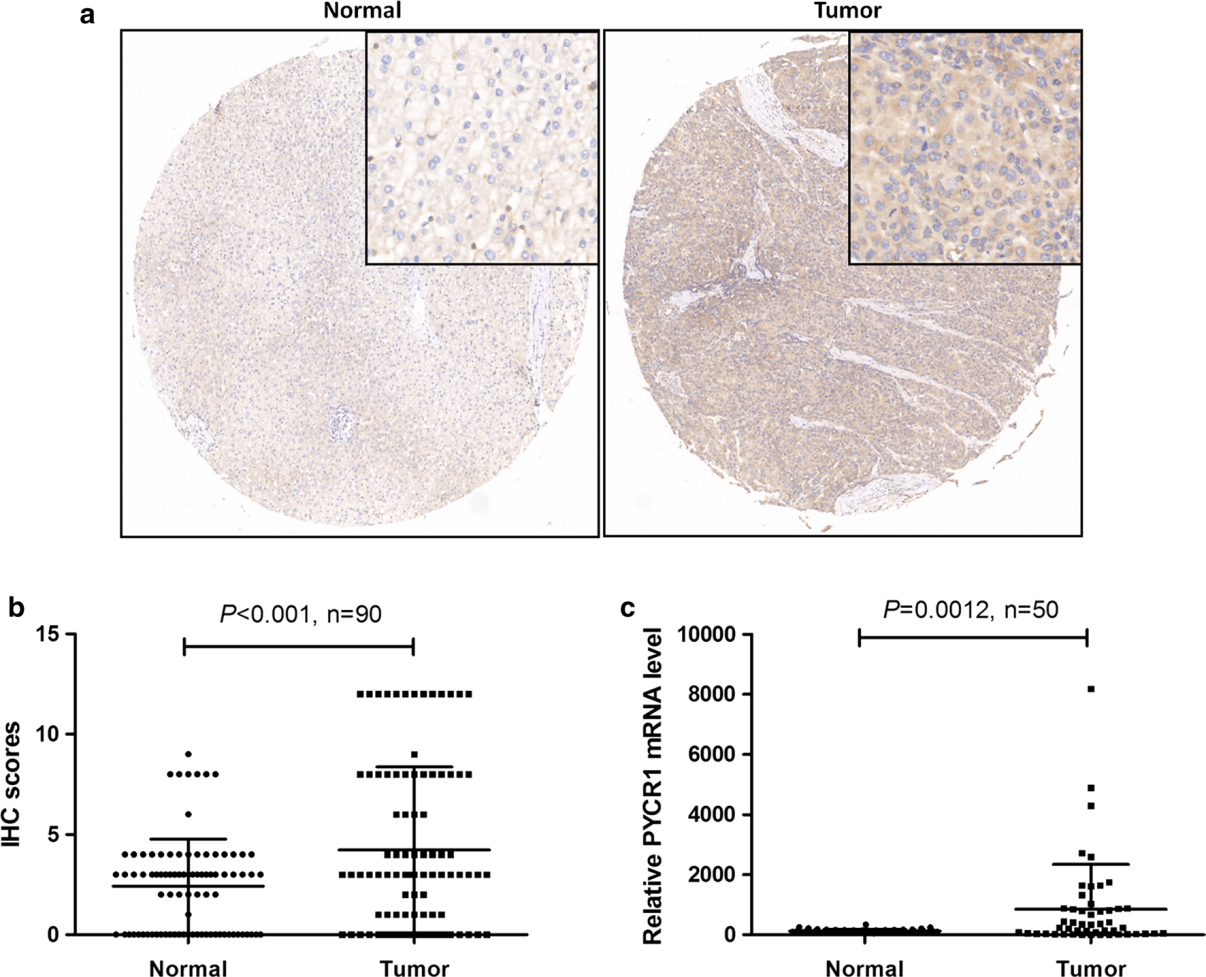
The expression level of PYCR1 in HCC patients. **a**, **b** The proein expression levels of PYCR1 in 90 pairs of HCC tumor and adjacent normal liver tissues were detected by IHC. PYCR1 levels were significantly upregulated in tumor tissues (*P *< 0.001). N = 90. **c** The mRNA expression levels of PYCR1 in 50 pairs of HCC tumor and adjacent normal liver tissues were obtained from TCGA database. PYCR1 levels were significantly upregulated in tumor tissues (*P *= 0.0012). N = 50. Values were expressed as mean ± standard deviation

To find out whether PYCR1 was dysregulated in HCC patients, we determined PYCR1 protein expression levels in tumor and adjacent normal liver tissues of HCC patients by IHC. Our data revealed that PYCR1 levels were higher expressed in tumor tissues than adjacent normal ones (Fig. 1a), and IHC scores were statistically significant higher in tumor tissues (*P *< 0.001, Fig. 1b). Furthermore, PYCR1 mRNA levels in 50 pairs of HCC tumor and adjacent normal liver tissues obtained from TCGA database were analyzed. As shown in Fig. 1c, PYCR1 levels were significantly upregulated in tumor tissues (*P *< 0.01). Both our data and TCGA data proved that PYCR1 was over expressed in HCC tumor tissues.

### PYCR1 interference inhibited cell proliferation and promoted cell apoptosis in vitro

**Fig. 2:**
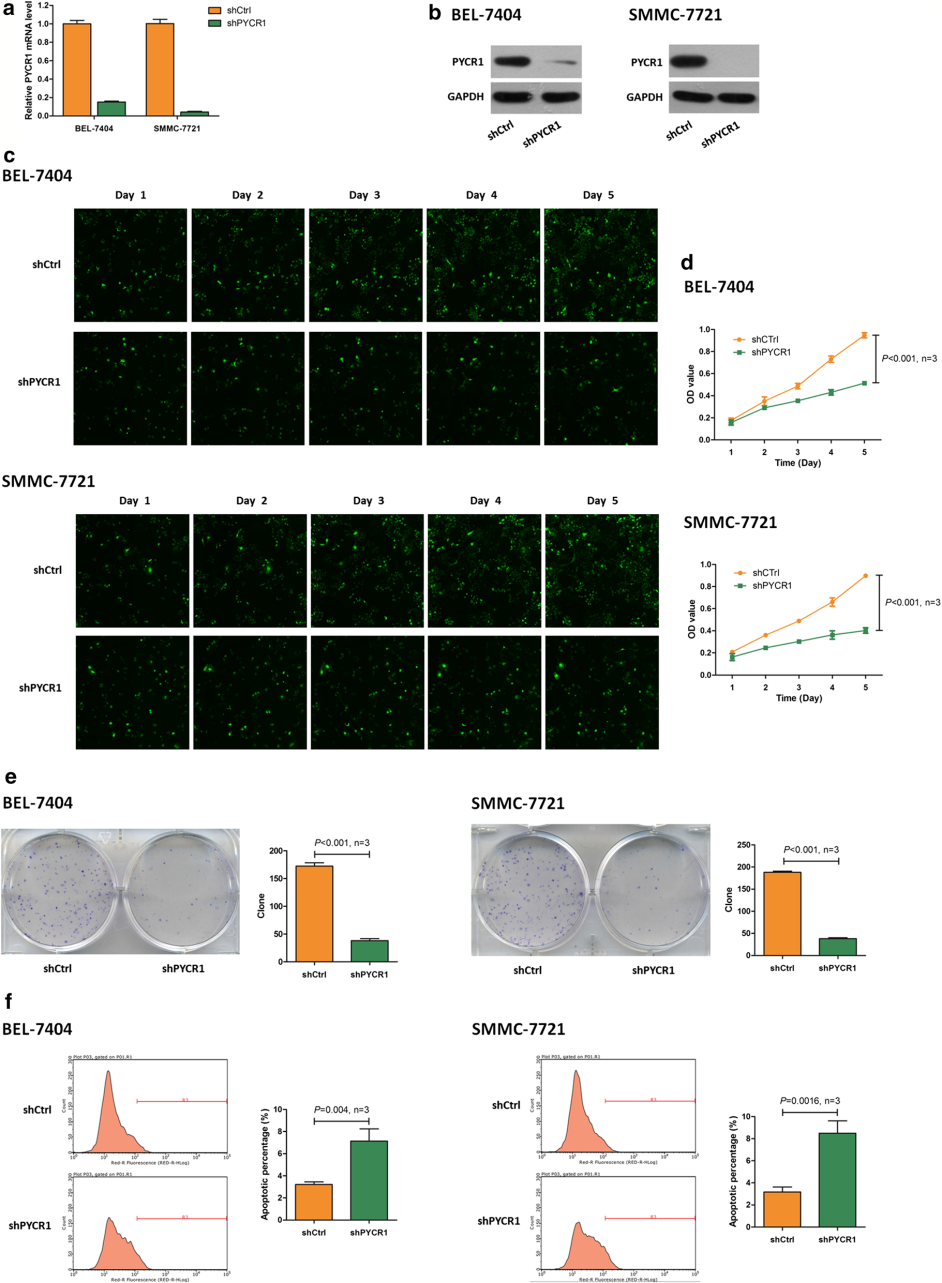
The cell proliferation and cell apoptosis influenced by PYCR1 interference in BEL-7404 and SMMC-7721 cells. **a**, **b** shPYCR1 group suppressed PYCR1 expression in both mRNA and protein levels. GAPDH was as the internal standard. **c** Celigo detection. PYCR1 interference significantly inhibited cell proliferation in both BEL-7404 and SMMC-7721 cells. **d** MTT assay. Cell growth was slower in shPYCR1 group than shCtrl group. On 5th day, the cell count was significantly smaller in shPYCR1 group than shCtrl group in both BEL-7404 and SMMC-7721 cells (*P *< 0.001). **e** Colony formation assay. PYCR1 interference significantly decreased the colony number in BEL-7404 and SMMC-7721 cells (*P *< 0.001). **f** Apoptosis assay. PYCR1 interference significantly increased the percentage of apoptosis cells in both BEL-7404 (*P *= 0.004) and SMMC-7721 cells (*P *= 0.0016). N = 3. Values were expressed as mean ± standard deviation

To find out whether PYCR1 influenced HCC tumorigenesis, we carried out PYCR1 interference by infecting with control lentivirus and shPYCR1 lentivirus in BEL-7404 and SMMC-7721 cells. Compared with controls, infected with shPYCR1 lentivirus led to suppressed PYCR1 expression in both mRNA and protein levels (Fig. 2a, b). Subsequently, Celigo and MTT results showed that cell growth was significantly slower in shPYCR1 group according to 5 days of detecting (*P *< 0.001, Fig. 2c, d). Similarly, colony formation result showed that the colony number was significantly smaller after PYCR1 interference (*P *< 0.001, Fig. 2e). Furthermore, in both BEL-7404 and SMMC-7721 cells, the percentage of apoptosis cells significantly increased in shPYCR1 group compared with control group (*P *< 0.01, Fig. 2f). These results indicated that PYCR1 interference inhibited cell proliferation and promoted cell apoptosis.

### PYCR1 interference suppressed tumor growth in vivo

**Fig. 3:**
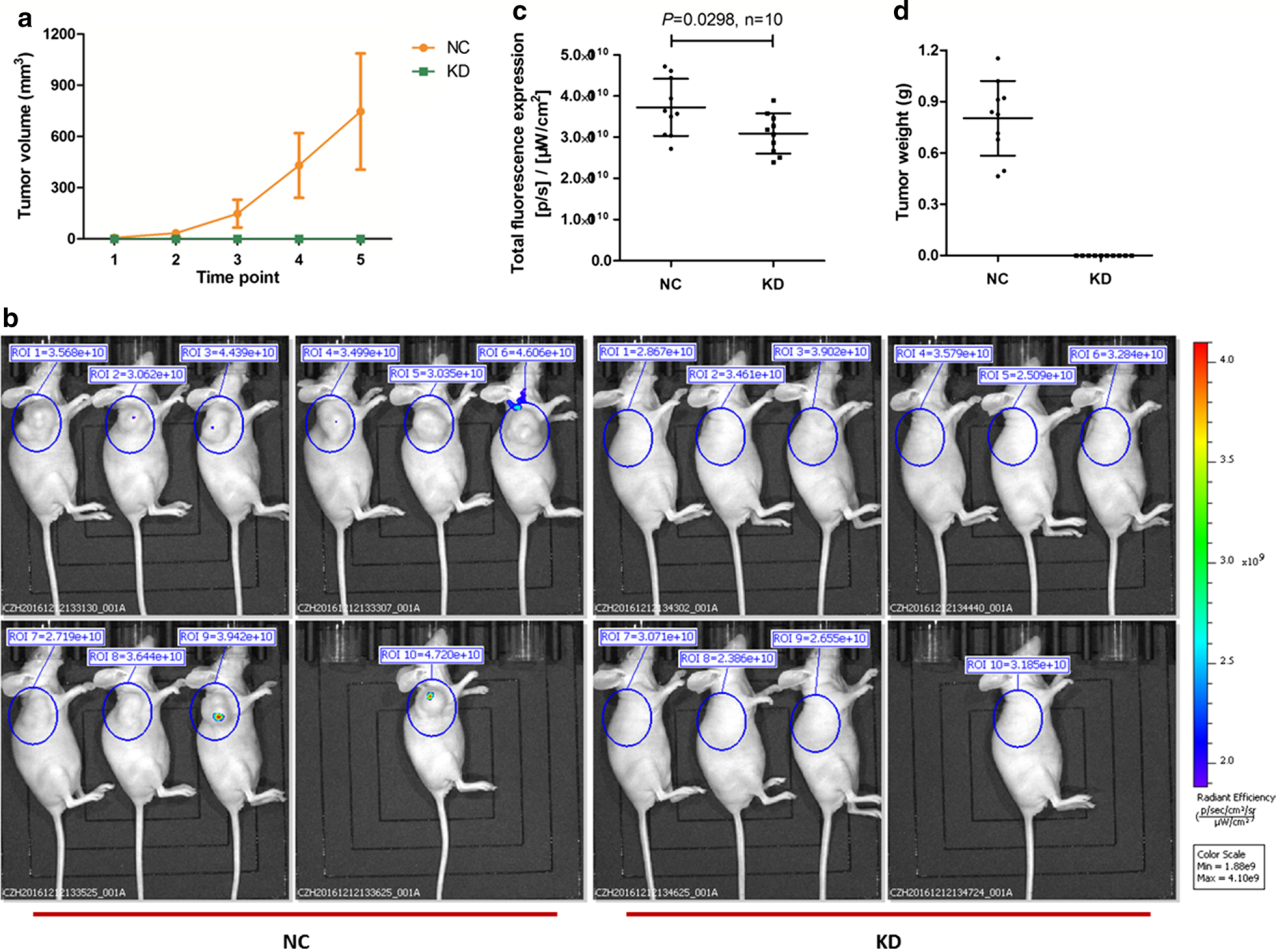
The effect of PYCR1 interference in vivo. **a** The change of tumor volume between NC group and KD group. After the diameter of tumor was larger than 5 mm, tumor volume was determined twice per week. Tumor volume = length * width^2^ * 0.52. **b** The bioluminescence imaging in NC group and KD group before sacrifice. **c** The scatter plot of total fluorescence expression in different groups. Compared with NC group, total fluorescence expression was significantly lower in KD group (*P *= 0.0298). **d** The tumor weight in NC group and KD group when sacrifice. Tumor weight was significantly increasing in NC group, while the tumors scarcely formed in KD group. N = 10. Values were expressed as mean ± standard deviation

To further validate the effect of PYCR1 in vivo, we next used a nude mouse xenograft assay with shCtrl and shPYCR1 SMMC-7721 cells. Tumor volume were significantly increasing with the passage of time in shCtrl treated NC group, while the tumors scarcely formed in shPYCR1 treated KD group (Fig. 3a). Before sacrifice, the growth of xenograft tumors was monitored by bioluminescence imaging. The pictures of nude mice in two groups were shown in Fig. 3b and total fluorescence expression was analyzed in Fig. 3c. Compared with NC group, total fluorescence expression was significantly lower in KD group (*P *< 0.05). After sacrifice, the tumors were weighed, and obvious tumors were formed in NC group while no tumors were observed in KD group (Fig. 3d). These results suggested that PYCR1 interference could obviously suppress tumor growth in vivo.

### PYCR1 interference regulated c-Jun N-terminal kinase/insulin receptor substrate 1 (JNK/IRS1) pathway in HCC cells

**Fig. 4:**
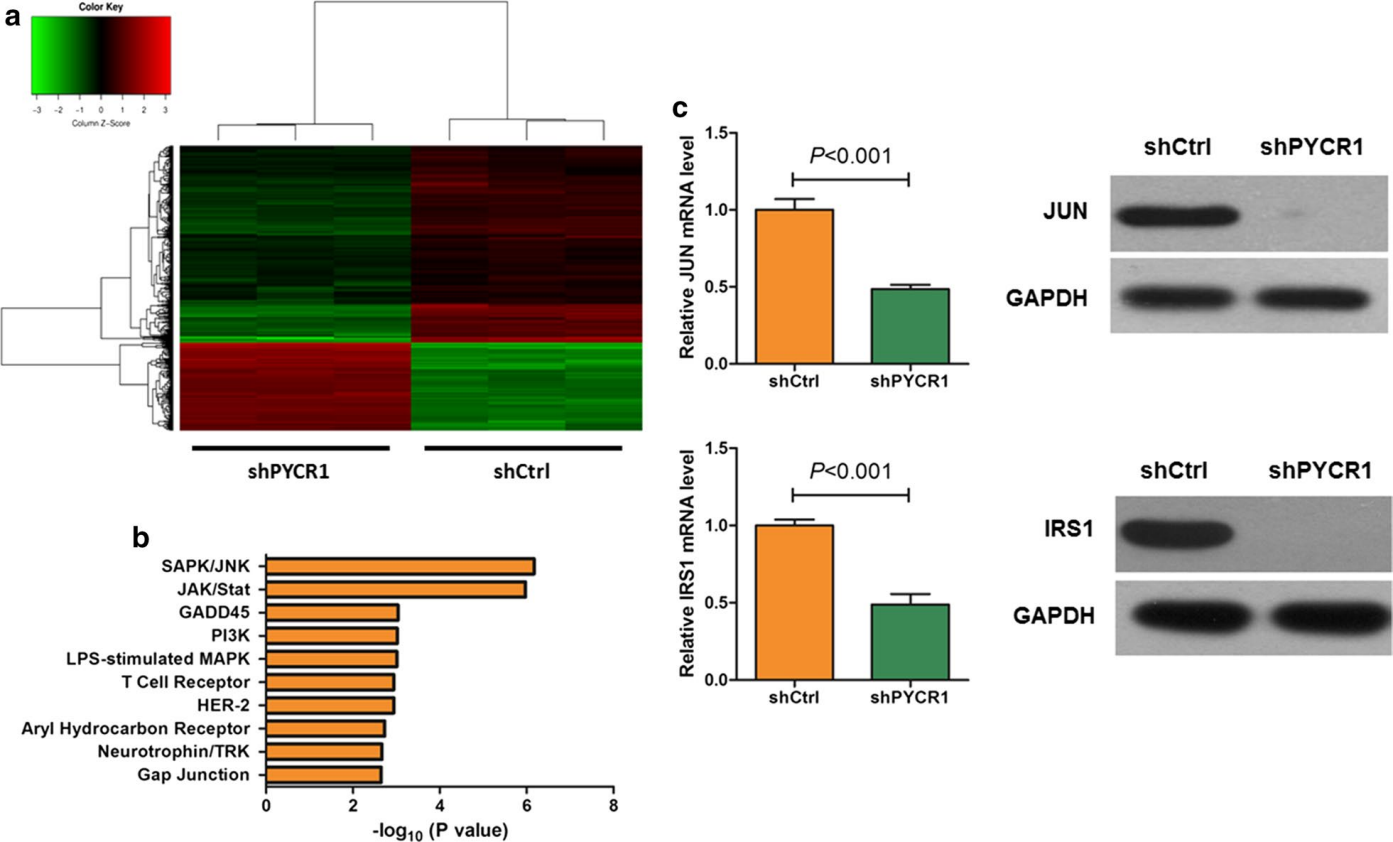
The JNK/IRS1 pathway influenced by PYCR1 interference. **a** The heatmap of different expressed genes between shCtrl group and shPYCR1 group. Red represents up-regulated genes, and green represents down-regulated genes. Upper tree structure is listed according to the sample characteristics, and left tree structure is listed according to the gene characteristics. There is a higher similarity between the adjacent samples or genes. **b** The significantly altered canonical pathway between shCtrl group and shPYCR1 group. The result showed that SAPK/JNK was the most significantly altered signaling pathway. **c** The mRNA and protein expressions of c-Jun and IRS1 were determined by qRT-PCR and western blot. Both c-Jun and IRS1 were significantly down-regulated by PYCR1 interference in mRNA and protein levels (*P *< 0.001). GAPDH was as the internal standard. N = 3. Values were expressed as mean ± standard deviation

To explore the mechanisms of PYCR1 interference inhibiting cell proliferation and promoting apoptosis in HCC cells, we detected the gene expression profiling of shCtrl group and shPYCR1 group in SMMC-7721 cells. Compared with shCtrl group, 173 genes were up-regulated in shGINS2 group, and 379 genes were down-regulated. As shown in Fig. 4a, red referred to genes up-regulated, and green referred to ones down-regulated. The significantly altered canonical pathway were identified by IPA, and it showed that the value of − log_10_ (*P* value) for SAPK/JNK signaling pathway was highest (Fig. 4b). Subsequently, JUN and the enzyme IRS1 were chosen to validate by qRT-PCR and western blot, which were significantly down-regulated in microarray analysis. As shown in Fig. 4c, the verification results of JUN and IRS1 were consistent with the results of gene expression profiling, and they were significantly down-regulated by PYCR1 interference in both mRNA and protein levels (*P *< 0.001), implying that PYCR1 might influence HCC cells proliferation and apoptosis by regulating JNK/IRS1 pathway.

## Discussion

In this study, we investigated the role of human PYCR1, so-far a poorly studied protein. PYCR1 is one of three human PYCR isoenzymes. PYCR catalyzes the final step in the conversion of Δ1-pyrroline-5-carboxylate (P5C) to proline with concomitant oxidation of NAD(P)H to NAD(P)^+^ [17]. In clinically, we analyzed PYCR1 protein and mRNA levels from 140 pairs of tumor and adjacent normal liver tissues of HCC patients, and found that PYCR1 levels were significantly up-regulated in HCC tumor tissues than adjacent normal liver tissues (Fig. 1). In vitro, after PYCR1 interference, cell growth was significantly slower via celigo and MTT assay, the colony number was significantly smaller, and the percentage of apoptosis cells significantly increased (Fig. 2). In vivo, PYCR1 interference could obviously suppress tumor growth in xenograft nude mice (Fig. 3). These results indicated that PYCR1 interference might influence the occurrence and development of HCC.

Further investigation found that SAPK/JNK signaling pathway was significantly altered after PYCR1 interference (Fig. 4b). C-Jun-N-terminal kinase (JNK) is a mitogen-activated protein kinase (MAPK) family member [19]. JNK signaling is associated with cell death, survival, proliferation and differentiation. JNK activity regulates various pathophysiologic processes, including steatosis, inflammation, and insulin resistance [20]. Notably, many researchers found that JNK pathway could be a crucial mediator of insulin resistance [21]. It has been validated that excessive JNK activation leads to suppression of insulin-gene expression and promotion of systemic insulin deficiency and pancreatic β cells dysfunction [22]. It is well known that insulin resistance has a close relationship with cancer. A meta-analysis of observational studies has revealed that insulin resistance is a significant risk factor for endometrial cancer [23]. It is widely accepted that diabetic patients have relatively increased cancer risk as well as worse cancer prognosis, in comparison with individuals without diabetes [24]. Therefore, molecules and pathways related insulin resistance, such as JNK signaling pathway, should be paid attention in the occurrence and development of cancer.

JNK was reported to drive insulin resistance via direct phosphorylation of insulin receptor substrate (IRS) [25], a family of adaptor proteins that are essential for insulin effects [26]. Our study confirmed that mRNA and protein expression levels of IRS1 were significantly down-regulated by PYCR1 interference (Fig. 4c). IRS1 is cytoplasmic substrate of the insulin receptor (INSR) and insulin-like growth factor 1 receptor (IGF1R) signaling pathways [27]. On one hand, IRS1 mediates glucose homeostasis as well as proliferative and anti-apoptotic function of insulin and IGF1 [28]. On the other hand, IRS1 also plays prominent roles in human malignancy and is activated in various human cancers [29, 30]. Phosphorylation of IRS1 serves to recruit downstream effectors leading to activation of the MAPK cascade, which promotes activation of the PI3K cascade and further causes increased protein and glycogen synthesis through the phosphorylation of the mammalian target of rapamycin (mTOR) and glycogen synthase kinase-3b (GSK3B) [31].

Many researchers thought that amino acid concentrations were associated to insulin resistance [32]. In the past years the attention has been focused on circulating branched chain amino acids (BCAA) that might play a role in promoting peripheral and hepatic insulin resistance [33–35]. Notably, elevated circulating levels of proline were found in subjects with insulin resistance [36]. As an important enzyme of proline biosynthesis, PYCR1 might influence HCC cell growth and survival via multiple ways, such as insulin resistance found in our study. Elevated proline might cause reduced β-cell mass by decreasing β-cell mitotic division and proliferation, and exposure to excess proline caused increased basal insulin secretion and impared glucose-stimulated insulin secretion [37].

## Conclusion

**Fig. 5:**
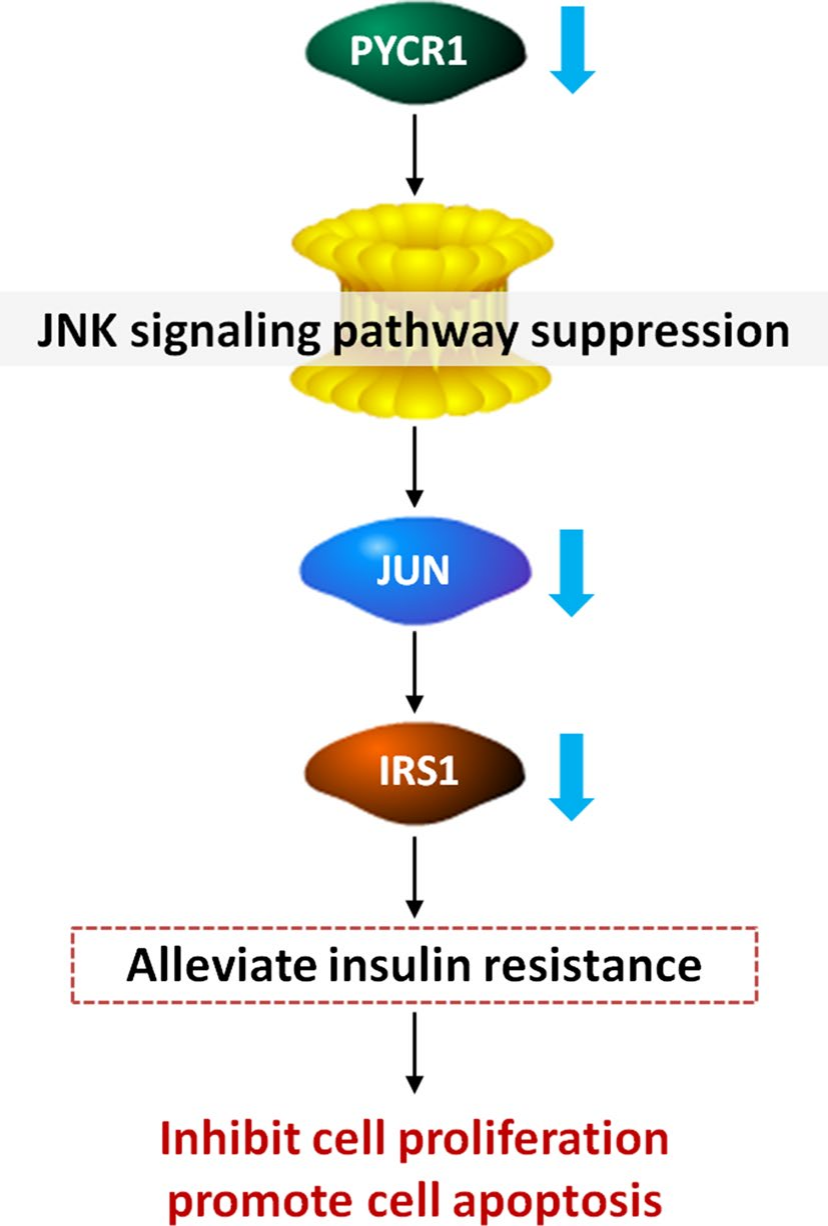
Proposed model of PYCR1 interference on HCC cell proliferation and apoptosis. PYCR1 interference could inhibit IRS1 expression and insulin resistance via JNK signaling pathway suppression, which subsequently inhibited HCC cell proliferation and promoted cell apoptosis

In conclusion, we report here that PYCR1 interference could inhibit cell proliferation and promote cell apoptosis in HCC, and the mechanisms might be involved in the regulation of JNK/IRS1 pathway (Fig. 5). Our study gains a new insight into the potential prominent role of PYCR1 in regulating insulin resistance of HCC. It will provide a drug target for HCC therapy and a potential biomarker for diagnosis or prognosis. However, the exploration of the specific targets of PYCR1 needs our further study.

## Data Availability

The datasets used and/or analyzed during the current study are available from the corresponding author on reasonable request.

## Abbreviations

BCA: bicinchoninic acid
BCAA: branched chain amino acids
GSK3B: glycogen synthase kinase-3b
HCC: hepatocellular carcinoma
IGF1R: insulin-like growth factor 1 receptor
INSR: insulin receptor
IRS: insulin receptor substrate
JNK/IRS1: c-Jun N-terminal kinase/insulin receptor substrate 1
MAPK: mitogen-activated protein kinase
mTOR: the mammalian target of rapamycin
NEAA: nonessential amino acids
P5C: pyrroline-5-carboxylate
PVDF: polyvinylidene difluoride
PYCR1: pyrroline-5-carboxylate reductase 1
qRT-PCR: quantitative real-time polymerase chain reaction
